# The genome sequence of pellitory-of-the-wall, *Parietaria judaica* L. (Urticaceae)

**DOI:** 10.12688/wellcomeopenres.23153.1

**Published:** 2024-10-17

**Authors:** Maarten J. M. Christenhusz, Royal Botanic Gardens Kew Genome Acquisition Lab, Darwin Tree of Life Barcoding collective, Plant Genome Sizing collective, Wellcome Sanger Institute Tree of Life Management, Samples and Laboratory team, Wellcome Sanger Institute Scientific Operations: Sequencing Operations, Wellcome Sanger Institute Tree of Life Core Informatics team, Tree of Life Core Informatics collective, Darwin Tree of Life Consortium

**Affiliations:** 1 Royal Botanic Gardens Kew, Richmond, England, UK; 2 Curtin University, Perth, Western Australia, Australia

**Keywords:** Parietaria judaica, pellitory-of-the-wall genome sequence, chromosomal, Rosales

## Abstract

We present a genome assembly from an individual *Parietaria judaica* (pellitory-of-the-wall; Tracheophyta; Magnoliopsida; Rosales; Urticaceae). The genome sequence is 538.7 megabases in span. Most of the assembly is scaffolded into 13 chromosomal pseudomolecules. The three mitochondrial genomes have lengths of 215.28, 107.63 and 112.60 kilobases, while the plastid genome assembly had a length of 152.63 kilobases. Gene annotation of this assembly on Ensembl identified 35,329 protein-coding genes.

## Species taxonomy

Eukaryota; Viridiplantae; Streptophyta; Streptophytina; Embryophyta; Tracheophyta; Euphyllophyta; Spermatophyta; Magnoliopsida; Mesangiospermae; eudicotyledons; Gunneridae; Pentapetalae; rosids; fabids; Rosales; Urticaceae; *Parietaria*; *Parietaria judaica* L., 1763 (NCBI:txid33127).

## Background

*Parietaria judaica* or pellitory-of-the-wall, also known as spreading pellitory or asthma weed, is a highly-branched perennial herb with stems that become woody at their base. *Parietaria* is Latin for ‘wall-dweller’ and the specific name *judaica* means ‘of Judaea’, a region in the Levant, from where the plant was first described ( Linnaeus & Strand, 1756). Growing up to about 60 cm, these plants are covered in hairs but lack the sting characteristic of other members of the Urticaceae family. Its alternate, simple leaves have entire margins, and the small whitish or pinkish flowers are formed in clusters in the leaf axils. Fruits are dark green achenes. *P. judaica* is an important host plant for caterpillars of several butterfly species, including the Red Admiral *Vanessa atalanta* Linnaeus, 1758 ( Lafranchis, 2019).

This plant is common throughout Europe, west and central Asia, and in north Africa, but is absent or rare in the colder north and on mountains at altitudes exceeding 1,000 m. *P. judaica* is commonly found in urban areas, growing in cracks in mortar, on rubble, rocks, cliffs, and hedge banks, usually in dry, sunny, and sheltered locations. In the UK, this herb is often found on abbey walls, which may be related to its use as a remedy for urinary diseases by medieval herbalists ( Stroh *et al.*, 2023).

*Parietaria judaica* flowers are wind pollinated and the copious amounts of irritant pollen can cause allergic reactions, including asthma ( Cancelliere *et al.*, 2020; Ciprandi *et al.*, 2018; Colombo *et al.*, 2003; Dorofeeva *et al.*, 2019; Stumvoll *et al.*, 2003). Seeds are easily transported by people, cars, pets, insects and other animals, and so it has become an invasive species in temperate regions of North America, Australia and New Zealand ( Bass & Bass, 1990; Kaufman, 1990), where it also causes pollen allergies, especially in urban areas.

We present the first complete genome of *Parietaria judaica*, which we hope will facilitate immunological studies on allergens in this species. The assembly of the genome into 13 chromosomal pseudomolecules is consistent with the previously published chromosome count for *P. judaica* of 2 *n* = 26 ( Stace *et al.*, 2019).

## Genome sequence report

**Figure 1.**
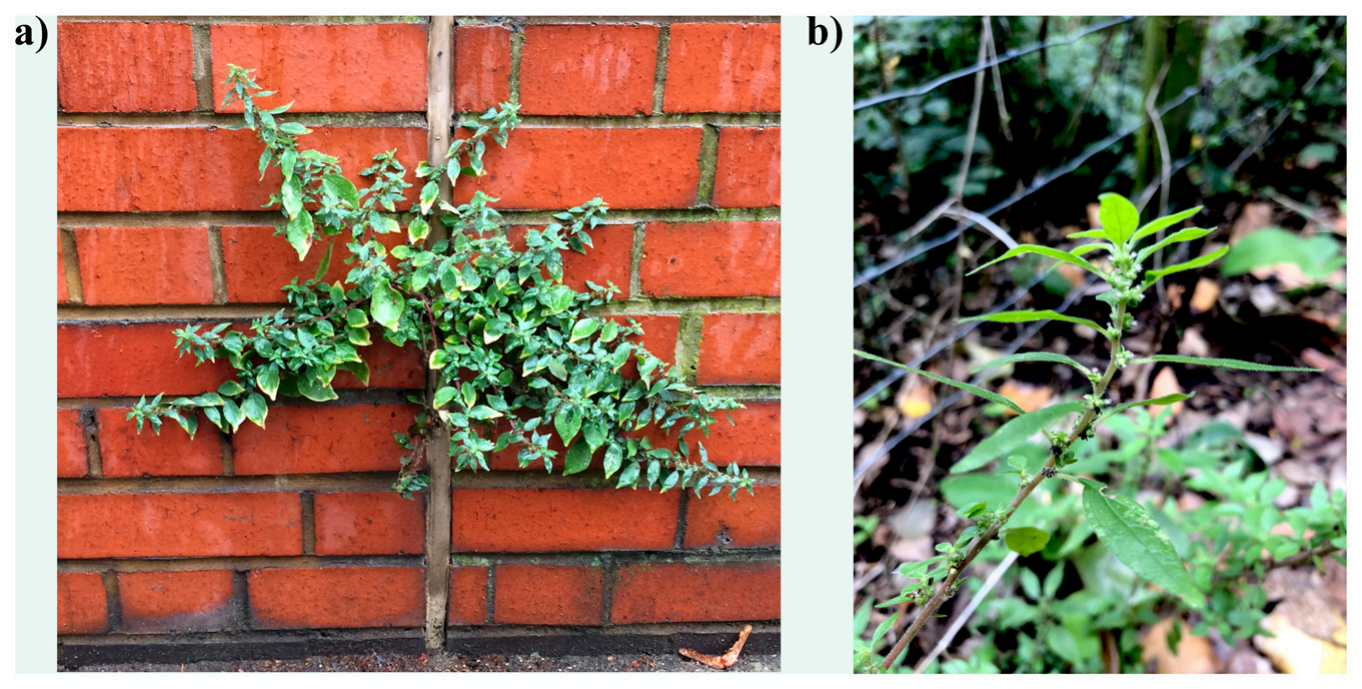
Photograph of the *Parietaria judaica* (drParJuda1). **a**) plant growing on a wall in Chelsea, London, UK. **b**) specimen at RBG Kew used for genome sequencing.

The genome of a specimen of *Parietaria judaica* ( Figure 1) was sequenced using Pacific Biosciences single-molecule HiFi long reads, generating a total of 18.72 Gb (gigabases) from 1.43 million reads, providing approximately 29-fold coverage. Using flow cytometry, the genome size (1C-value) was estimated to be 0.77 pg, equivalent to 750 Mb. Primary assembly contigs were scaffolded with chromosome conformation Hi-C data, which produced 115.06 Gb from 761.99 million reads, yielding an approximate coverage of 214-fold. Specimen and sequencing information is summarised in Table 1.

**Table 1.** Specimen and sequencing data for *Parietaria judaica*.

Project information			
**Study title**	*Parietaria judaica*		
**Umbrella BioProject**	PRJEB52661		
**Species**	*Parietaria judaica*		
**BioSample**	SAMEA7522182		
**NCBI taxonomy ID**	33127		
Specimen information			
**Technology**	**ToLID**	**BioSample accession**	**Organism part**
**PacBio long read sequencing**	drParJuda1	SAMEA7522280	leaf
**Hi-C sequencing**	drParJuda1	SAMEA7522299	leaf
**RNA sequencing**	drParJuda1	SAMEA7522297	leaf
Sequencing information			
**Platform**	**Run accession**	**Read count**	**Base count (Gb)**
**Illumina NovaSeq 6000 (Hi-C)**	ERR9710928	7.62e+08	115.06
**PacBio Sequel IIe**	ERR9709341	7.36e+05	10.24
**PacBio Sequel IIe**	ERR9709342	6.89e+05	8.47
**Illumina NovaSeq 6000 (RNA)**	ERR11641095	7.54e+07	11.38

**Figure 2.**
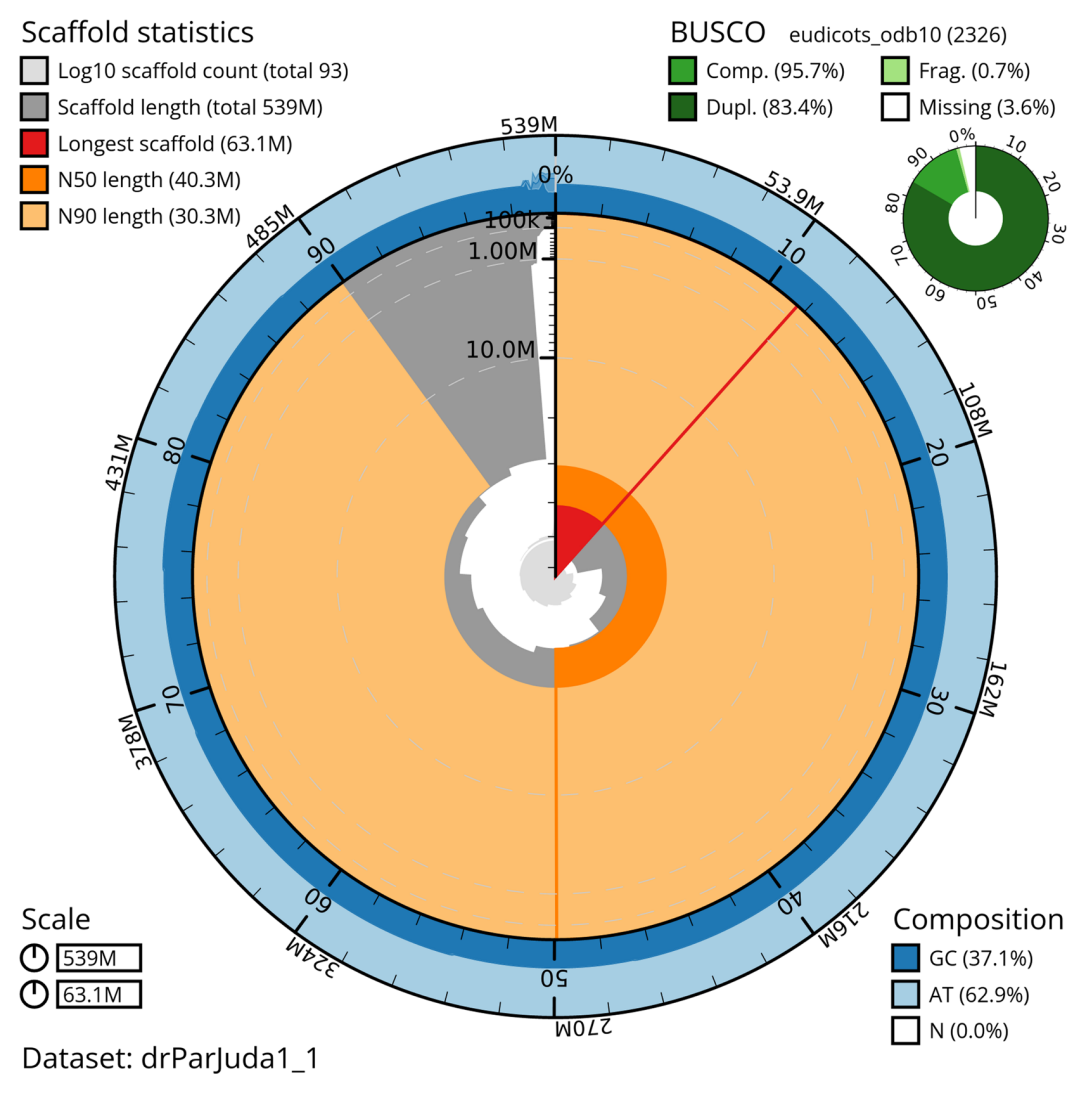
Genome assembly of *Parietaria judaica*, drParJuda1.1: metrics. The BlobToolKit Snailplot shows N50 metrics and BUSCO gene completeness. The main plot is divided into 1,000 size-ordered bins around the circumference with each bin representing 0.1% of the 539,320,906 bp assembly. The distribution of scaffold lengths is shown in dark grey with the plot radius scaled to the longest scaffold present in the assembly (63,050,874 bp, shown in red). Orange and pale-orange arcs show the N50 and N90 scaffold lengths (40,273,085 and 30,341,377 bp), respectively. The pale grey spiral shows the cumulative scaffold count on a log scale with white scale lines showing successive orders of magnitude. The blue and pale-blue area around the outside of the plot shows the distribution of GC, AT and N percentages in the same bins as the inner plot. A summary of complete, fragmented, duplicated and missing BUSCO genes in the eudicots_odb10 set is shown in the top right. An interactive version of this figure is available at https://blobtoolkit.genomehubs.org/view/drParJuda1_1/dataset/drParJuda1_1/snail.

**Figure 3.**
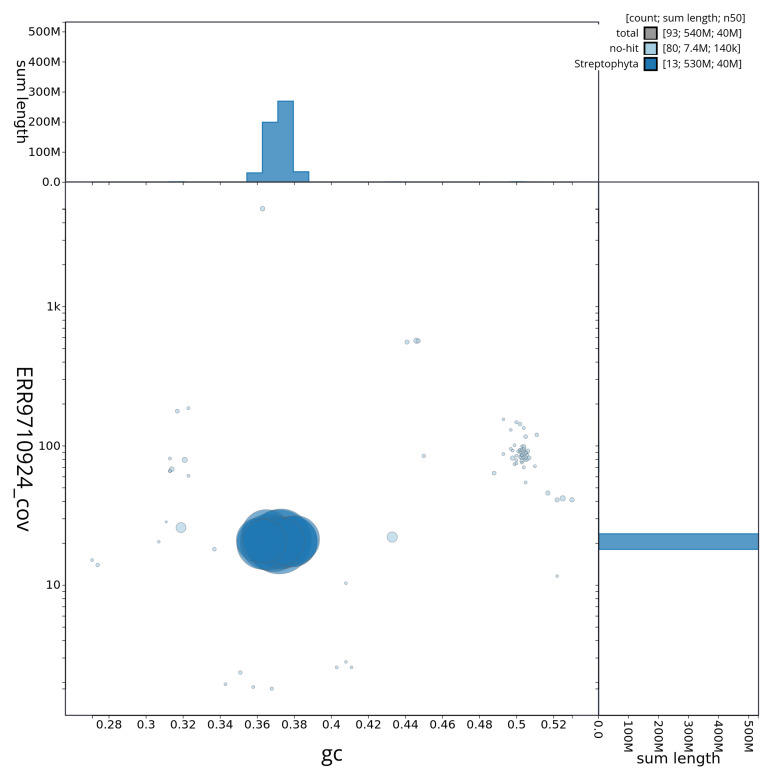
Blob plot of base coverage against GC proportion for sequences in the assembly drParJuda1.1. Scaffolds are coloured by phylum. Circles are sized in proportion to scaffold length. Histograms show the distribution of scaffold length sum along each axis. An interactive version of this figure is available at https://blobtoolkit.genomehubs.org/view/drParJuda1_1/dataset/drParJuda1_1/blob.

**Figure 4.**
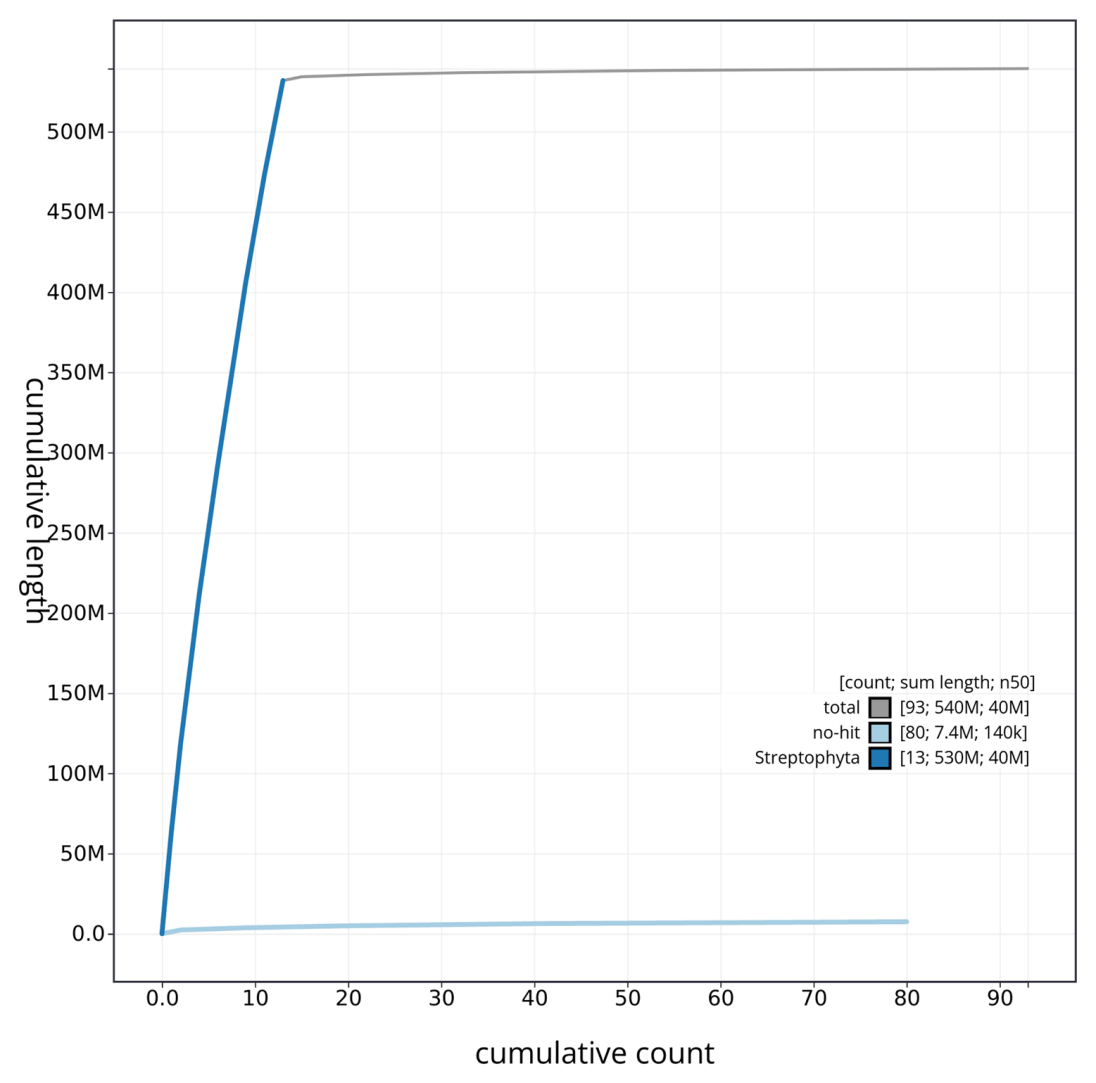
Genome assembly of *Parietaria judaica*, drParJuda1.1: BlobToolKit cumulative sequence plot. The grey line shows cumulative length for all scaffolds. Coloured lines show cumulative lengths of scaffolds assigned to each phylum using the buscogenes taxrule. An interactive version of this figure is available at https://blobtoolkit.genomehubs.org/view/drParJuda1_1/dataset/drParJuda1_1/cumulative.

**Figure 5.**
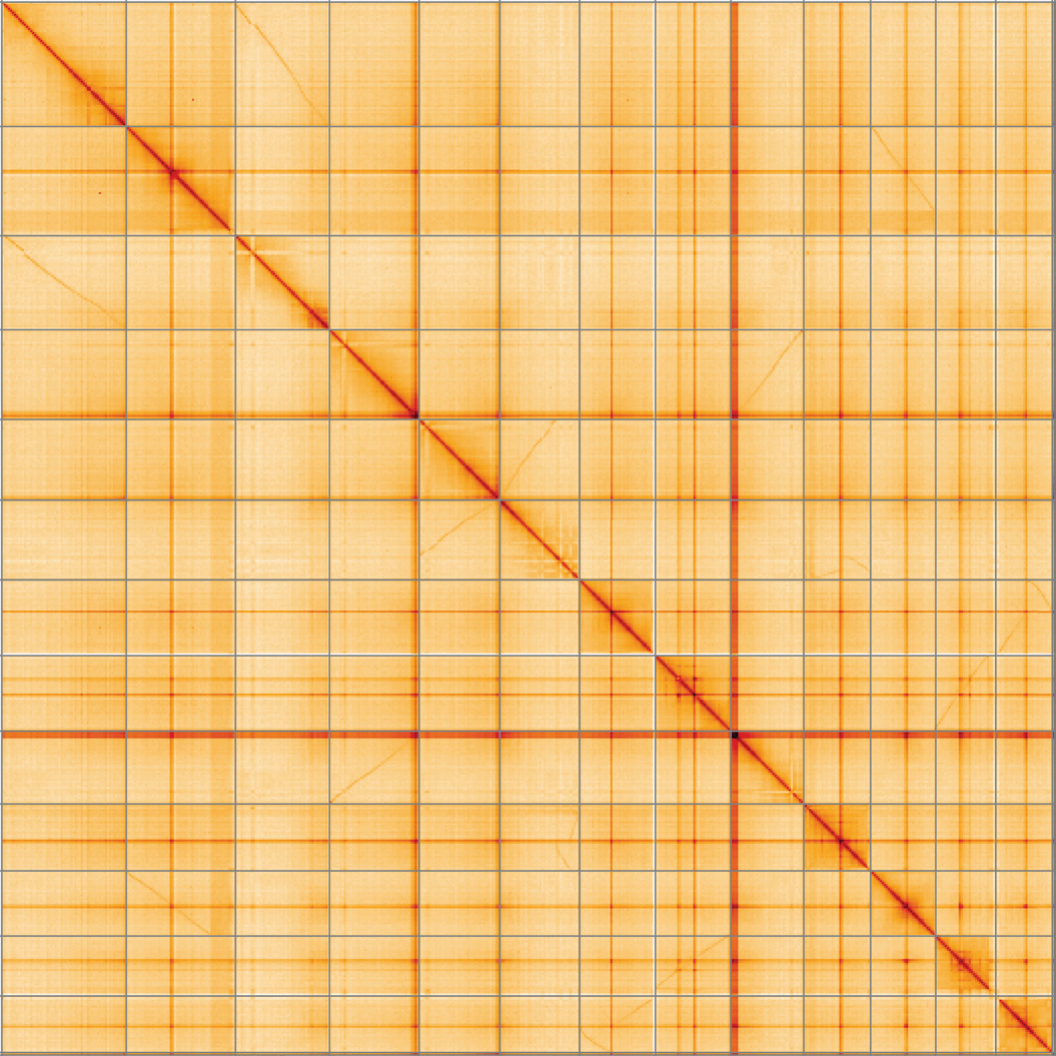
Genome assembly of *Parietaria judaica*, drParJuda1.1, Hi-C contact map of the drParJuda1.1. assembly, visualised using HiGlass. Chromosomes are shown in order of size from left to right and top to bottom. An interactive version of this figure may be viewed at https://genome-note-higlass.tol.sanger.ac.uk/l/?d=e2CzejAaRtKF0I-Mg-711g.

Manual assembly curation corrected 12 missing joins or mis-joins and removed two haplotypic duplications, reducing the scaffold number by 2.11%, and increasing the scaffold N50 by 0.99%. The final assembly has a total length of 538.70 Mb in 89 sequence scaffolds with a scaffold N50 of 40.3 Mb ( Table 2) with 36 gaps. The snail plot in Figure 2 provides a summary of the assembly statistics, while the distribution of assembly scaffolds on GC proportion and coverage is shown in Figure 3. The cumulative assembly plot in Figure 4 shows curves for subsets of scaffolds assigned to different phyla. Most (98.77%) of the assembly sequence was assigned to 13 chromosomal-level scaffolds. Chromosome-scale scaffolds confirmed by the Hi-C data are named in order of size ( Figure 5; Table 3). While not fully phased, the assembly deposited is of one haplotype. Contigs corresponding to the second haplotype have also been deposited. The mitochondrial and plastid genomes were also assembled and can be found as contigs within the multifasta file of the genome submission.

**Table 2.** Genome assembly data for *Parietaria judaica*, drParJuda1.1.

Genome assembly		
Assembly name	drParJuda1.1	
Assembly accession	GCA_954871525.1	
*Accession of alternate haplotype*	*GCA_954870795.1*	
Span (Mb)	538.70	
Number of contigs	129	
Contig N50 length (Mb)	16.6	
Number of scaffolds	89	
Scaffold N50 length (Mb)	40.3	
Longest scaffold (Mb)	63.27	
Assembly metrics *		*Benchmark*
Consensus quality (QV)	55.2	*≥ 50*
*k*-mer completeness	99.99%	*≥ 95%*
BUSCO **	C:95.7%[S:12.3%,D:83.4%], F:0.7%,M:3.6%,n:2,326	*C ≥ 95%*
Percentage of assembly mapped to chromosomes	98.77%	*≥ 95%*
Organelles	Mitochondrial sequences: 215.28, 107.63 and 112.60 kb; plastid genome: 152.63 kb	*complete single * *alleles*
Genome annotation at Ensembl		
Number of protein-coding genes	35,329	
Number of non-coding genes	11,120	
Number of gene transcripts	56,410	

* Assembly metric benchmarks are adapted from column VGP-2020 of “Table 1: Proposed standards and metrics for defining genome assembly quality” from Rhie *et al.* (2021). ** BUSCO scores based on the eudicots_odb10 BUSCO set using version 5.4.3. C = complete [S = single copy, D = duplicated], F = fragmented, M = missing, n = number of orthologues in comparison. A full set of BUSCO scores is available at https://blobtoolkit.genomehubs.org/view/drParJuda1_1/dataset/drParJuda1_1/busco.

**Table 3.** Chromosomal pseudomolecules in the genome assembly of *Parietaria judaica*, drParJuda1.

INSDC accession	Name	Length (Mb)	GC%
OX940860.1	1	63.05	37.0
OX940861.1	2	55.3	37.5
OX940862.1	3	47.61	36.5
OX940863.1	4	45.37	37.0
OX940864.1	5	40.88	37.0
OX940865.1	6	40.27	36.5
OX940866.1	7	38.37	38.0
OX940867.1	8	38.29	38.0
OX940868.1	9	36.87	36.5
OX940869.1	10	33.9	38.0
OX940870.1	11	33.01	37.5
OX940871.1	12	30.34	36.0
OX940872.1	13	28.6	36.5
OX940876.1	Pltd	0.15	36.5
OX940873.1	MT1	0.22	44.5
OX940874.1	MT2	0.11	44.0
OX940875.1	MT3	0.11	44.5

The estimated Quality Value (QV) of the final assembly is 55.2 with *k*-mer completeness of 99.99%, and the assembly has a BUSCO v5.4.3 completeness of 95.7% (single = 12.3%, duplicated = 83.4%), using the eudicots_odb10 reference set ( *n* = 2,326).

Metadata for specimens, BOLD barcode results, spectra estimates, sequencing runs, contaminants and pre-curation assembly statistics are given at https://links.tol.sanger.ac.uk/species/33127.

## Genome annotation report

The *Parietaria judaica* genome assembly (GCA_954871525.1) was annotated at the European Bioinformatics Institute (EBI) on Ensembl Rapid Release. The resulting annotation includes 56,410 transcribed mRNAs from 35,329 protein-coding and 11,120 non-coding genes ( Table 2; https://rapid.ensembl.org/Parietaria_judaica_GCA_954871525.1/Info/Index). The average transcript length is 2,556.68. There are 1.21 coding transcripts per gene and 4.98 exons per transcript.

## Methods

### Sample acquisition, DNA barcoding and genome size estimation

A specimen of *Parietaria judaica* (specimen ID KDTOL10061, ToLID drParJuda1) was collected from the Royal Botanic Gardens, Kew, Richmond, Surrey, UK (latitude 51.48, longitude –0.30) on 2020-08-26. The specimen was collected and identified by Maarten Christenhusz (Royal Botanic Gardens, Kew), and then frozen at –80 °C. The herbarium specimen of the sequenced plant, *Christenhusz 9057*, is deposited at the herbarium of RBG Kew (K) (K001400703).

The initial species identification was verified by an additional DNA barcoding process according to the framework developed by Twyford *et al.* (2024). Part of the plant specimen was preserved in silica gel desiccant. A DNA extraction from the dried plant was amplified by PCR for standard barcode markers, with the amplicons sequenced and compared to public sequence databases including GenBank and the Barcode of Life Database (BOLD). The barcode sequences for this specimen are openly available on BOLD ( Ratnasingham & Hebert, 2007). Following whole genome sequence generation, DNA barcodes were also used alongside the initial barcoding data for sample tracking through the genome production pipeline at the Wellcome Sanger Institute ( Twyford *et al.*, 2024). The standard operating procedures for the Darwin Tree of Life barcoding have been deposited on protocols.io ( Beasley *et al.*, 2023).

The genome size was estimated by flow cytometry using the fluorochrome propidium iodide and following the ‘one-step’ method as outlined in Pellicer *et al.* (2021). For this species, the General Purpose Buffer (GPB) supplemented with 3% PVP and 0.08% (v/v) beta-mercaptoethanol was used for isolation of nuclei ( Loureiro *et al.*, 2007), and the internal calibration standard was *Solanum lycopersicum* ‘Stupiké polní rané’ with an assumed 1C-value of 968 Mb ( Doležel *et al.*, 2007).

### Nucleic acid extraction

The workflow for high molecular weight (HMW) DNA extraction at the Wellcome Sanger Institute (WSI) Tree of Life Core Laboratory includes a sequence of core procedures: sample preparation and homogenisation, DNA extraction, fragmentation and purification. Detailed protocols are available on protocols.io ( Denton *et al.*, 2023). The drParJuda1 sample was weighed and dissected on dry ice ( Jay *et al.*, 2023), and leaf tissue was cryogenically disrupted using the Covaris cryoPREP ^®^ Automated Dry Pulverizer ( Narváez-Gómez *et al.*, 2023).

HMW DNA was extracted using the Automated Plant MagAttract v1 protocol ( Sheerin *et al.*, 2023). HMW DNA was sheared into an average fragment size of 12–20 kb in a Megaruptor 3 system ( Todorovic *et al.*, 2023). Sheared DNA was purified by solid-phase reversible immobilisation, using AMPure PB beads to eliminate shorter fragments and concentrate the DN ( Strickland *et al.*, 2023). The concentration of the sheared and purified DNA was assessed using a Nanodrop spectrophotometer and Qubit Fluorometer and Qubit dsDNA High Sensitivity Assay kit. Fragment size distribution was evaluated by running the sample on the FemtoPulse system.

RNA was extracted from leaf tissue of drParJuda1 in the Tree of Life Laboratory at the WSI using the RNA Extraction: Automated MagMax™ *mir*Vana protocol ( do Amaral *et al.*, 2023). The RNA concentration was assessed using a Nanodrop spectrophotometer and a Qubit Fluorometer using the Qubit RNA Broad-Range Assay kit. Analysis of the integrity of the RNA was done using the Agilent RNA 6000 Pico Kit and Eukaryotic Total RNA assay.

### Hi-C preparation

Hi-C data were generated from drParJuda1 leaf tissue using the Arima-HiC v2 kit at the WSI Scientific Operations core. Tissue was finely ground using cryoPREP and then subjected to nuclei isolation using a modified protocol of the Qiagen QProteome Kit. After isolation, the nuclei were fixed, and the DNA crosslinked using a 37% formaldehyde solution. The crosslinked DNA was then digested using the restriction enzyme master mix. The 5’-overhangs were then filled in and labelled with biotinylated nucleotides and proximally ligated. An overnight incubation was carried out for enzymes to digest remaining proteins and for crosslinks to reverse. A clean up was performed with SPRIselect beads prior to library preparation. DNA concentration was quantified using the Qubit Fluorometer v2.0 and Qubit HS Assay Kit according to the manufacturer’s instructions.

### Library preparation and sequencing

Library preparation and sequencing were performed at the WSI Scientific Operations core. Pacific Biosciences HiFi circular consensus DNA sequencing libraries were prepared using the PacBio Express Template Preparation Kit v2.0 (Pacific Biosciences, California, USA) as per the manufacturer’s instructions. The kit includes the reagents required for removal of single-strand overhangs, DNA damage repair, end repair/A-tailing, adapter ligation, and nuclease treatment. Library preparation also included a library purification step using 0.8X AMPure PB beads and a size selection step to remove templates < 3 kb using AMPure PB modified SPRI. Samples were sequenced using the Sequel IIe system (Pacific Biosciences, California, USA). The concentration of the library loaded onto the Sequel IIe was within the manufacturer's recommended loading concentration range of 40–100 pM. The SMRT link software, a PacBio web-based end-to-end workflow manager, was used to set-up and monitor the run, as well as perform primary and secondary analysis of the data upon completion.

Poly(A) RNA-Seq libraries were constructed using the NEB Ultra II RNA Library Prep kit, following the manufacturer’s instructions. RNA sequencing was performed on the Illumina NovaSeq 6000 instrument.

For Hi-C library preparation, DNA was fragmented to a size of 400 to 600 bp using a Covaris E220 sonicator. The DNA was then enriched, barcoded, and amplified using the NEBNext Ultra II DNA Library Prep Kit following manufacturers’ instructions. The Hi-C sequencing was performed using paired-end sequencing with a read length of 150 bp on an Illumina NovaSeq 6000 instrument.

### Genome assembly, curation and evaluation

***Assembly***

Assembly was carried out with Hifiasm ( Cheng *et al.*, 2021) and haplotypic duplication was identified and removed with purge_dups ( Guan *et al.*, 2020). The Hi-C reads were mapped to the primary contigs using bwa-mem2 ( Vasimuddin *et al.*, 2019). The contigs were further scaffolded using the provided Hi-C data ( Rao *et al.*, 2014) in YaHS ( Zhou *et al.*, 2023) using the --break option. The scaffolded assemblies were evaluated using Gfastats ( Formenti *et al.*, 2022), BUSCO ( Manni *et al.*, 2021) and MERQURY.FK ( Rhie *et al.*, 2020).

The mitochondrial and plastid genomes were assembled using MBG ( Rautiainen & Marschall, 2021) from PacBio HiFi reads mapping to related genomes. Representative circular sequences were selected for each from the graph based on read coverage.

***Curation***

The assembly was checked for contamination and corrected using the gEVAL system ( Chow *et al.*, 2016) as described previously ( Howe *et al.*, 2021). Manual curation was performed using gEVAL, HiGlass ( Kerpedjiev *et al.*, 2018) and PretextView ( Harry, 2022). Any identified contamination, missed joins, and mis-joins were corrected, and duplicate sequences were tagged and removed. The process is documented at https://gitlab.com/wtsi-grit/rapid-curation (article in preparation).

***Evaluation of final assembly***

A Hi-C map for the final assembly was produced using bwa-mem2 ( Vasimuddin *et al.*, 2019) in the Cooler file format ( Abdennur & Mirny, 2020). To assess the assembly metrics, the *k*-mer completeness and QV consensus quality values were calculated in Merqury ( Rhie *et al.*, 2020). This work was done using the “sanger-tol/readmapping” ( Surana *et al.*, 2023a) and “sanger-tol/genomenote” ( Surana *et al.*, 2023b) pipelines. The genome assembly and evaluation pipelines were developed using nf-core tooling ( Ewels *et al.*, 2020) and MultiQC ( Ewels *et al.*, 2016), relying on the Conda package manager, the Bioconda initiative ( Grüning *et al.*, 2018), the Biocontainers infrastructure ( da Veiga Leprevost *et al.*, 2017), as well as the Docker ( Merkel, 2014) and Singularity ( Kurtzer *et al.*, 2017) containerisation solutions.

The genome was also analysed within the BlobToolKit environment ( Challis *et al.*, 2020) and BUSCO scores ( Manni *et al.*, 2021) were calculated.

Table 4 contains a list of relevant software tool versions and sources.

**Table 4.** Software tools: versions and sources.

Software tool	Version	Source
BlobToolKit	4.1.7	https://github.com/blobtoolkit/blobtoolkit
BUSCO	5.3.2	https://gitlab.com/ezlab/busco
bwa-mem2	2.2.1	https://github.com/bwa-mem2/bwa-mem2
Cooler	0.8.11	https://github.com/open2c/cooler
gEVAL	N/A	https://geval.org.uk/
Gfastats	1.3.6	https://github.com/vgl-hub/gfastats
Hifiasm	0.15.3-r339	https://github.com/chhylp123/hifiasm
HiGlass	1.11.6	https://github.com/higlass/higlass
MBG	-	https://github.com/maickrau/MBG
Merqury	MerquryFK	https://github.com/thegenemyers/MERQURY.FK
PretextView	0.2	https://github.com/wtsi-hpag/PretextView
purge_dups	1.2.3	https://github.com/dfguan/purge_dups
sanger-tol/genomenote	v1.0	https://github.com/sanger-tol/genomenote
sanger-tol/readmapping	1.1.0	https://github.com/sanger-tol/readmapping/tree/1.1.0
YaHS	yahs-1.1.91eebc2	https://github.com/c-zhou/yahs

### Wellcome Sanger Institute – Legal and Governance

The materials that have contributed to this genome note have been supplied by a Darwin Tree of Life Partner. The submission of materials by a Darwin Tree of Life Partner is subject to the **‘Darwin Tree of Life Project Sampling Code of Practice’** which can be found in full on the Darwin Tree of Life website here. By agreeing with and signing up to the Sampling Code of Practice, the Darwin Tree of Life Partner agrees they will meet the legal and ethical requirements and standards set out within this document in respect of all samples acquired for, and supplied to, the Darwin Tree of Life Project.

Further, the Wellcome Sanger Institute employs a process whereby due diligence is carried out proportionate to the nature of the materials themselves, and the circumstances under which they have been/are to be collected and provided for use. The purpose of this is to address and mitigate any potential legal and/or ethical implications of receipt and use of the materials as part of the research project, and to ensure that in doing so we align with best practice wherever possible. The overarching areas of consideration are:

•   Ethical review of provenance and sourcing of the material

•   Legality of collection, transfer and use (national and international)

Each transfer of samples is further undertaken according to a Research Collaboration Agreement or Material Transfer Agreement entered into by the Darwin Tree of Life Partner, Genome Research Limited (operating as the Wellcome Sanger Institute), and in some circumstances other Darwin Tree of Life collaborators.

## Data Availability

European Nucleotide Archive: *Parietaria judaica*. Accession number PRJEB52661; https://identifiers.org/ena.embl/PRJEB52661 ( Wellcome Sanger Institute, 2023). The genome sequence is released openly for reuse. The *Parietaria judaica* genome sequencing initiative is part of the Darwin Tree of Life (DToL) project. All raw sequence data and the assembly have been deposited in INSDC databases. The genome will be annotated using available RNA-Seq data and presented through the Ensembl pipeline at the European Bioinformatics Institute. Raw data and assembly accession identifiers are reported in Table 1.

## Author information

Members of the Royal Botanic Gardens Kew Genome Acquisition Lab are listed here: https://doi.org/10.5281/zenodo.2625079.

Members of the Plant Genome Sizing collective are listed here: https://doi.org/10.5281/zenodo.7994306.

Members of the Darwin Tree of Life Barcoding collective are listed here: https://doi.org/10.5281/zenodo.12158331.

Members of the Wellcome Sanger Institute Tree of Life Management, Samples and Laboratory team are listed here: https://doi.org/10.5281/zenodo.12162482.

Members of Wellcome Sanger Institute Scientific Operations: Sequencing Operations are listed here: https://doi.org/10.5281/zenodo.12165051.

Members of the Wellcome Sanger Institute Tree of Life Core Informatics team are listed here: https://doi.org/10.5281/zenodo.12160324.

Members of the Tree of Life Core Informatics collective are listed here: https://doi.org/10.5281/zenodo.12205391.

Members of the Darwin Tree of Life Consortium are listed here: https://doi.org/10.5281/zenodo.4783558.
