# Supplementary material for: The genome sequence of pellitory-of-the-wall, Parietaria judaica L. (Urticaceae)
Source: Wellcome Open Res. 2024 Oct 17;9:608. [Version 1] doi: 10.12688/wellcomeopenres.23153.1 (PMC12514444; doi:10.12688/wellcomeopenres.23153.1)
Supplement: Peer review history

# Reviewer report: Reviewer response for version 1

**DOI:** 10.21956/wellcomeopenres.25497.r126793

**Published:** 2025-11-05

**Authors:** Abhisek Chakraborty

**Affiliations:** 1 Johannes Gutenberg Universitat Mainz Institut fur Organismische und Molekulare Evolutionsbiologie (Ringgold ID: 163392), Mainz, Rhineland-Palatinate, Germany; 2 Indian Institute of Science Education and Research Bhopal (IISERB), Bhopal, Madhya Pradesh, India

The authors presented the first complete genome of *Parietaria judaica*, which will be valuable for the research community. However, I have the following concerns for this data note –

1. Please discuss the reasons for the presence of such a high fraction (83.4%) of duplicated BUSCO genes in the genome assembly. Also, the assembled genome size is much smaller than the estimated genome size.
2. I feel that the genome annotation results were not described in details. For example, the genomic repeat content, the completeness of the coding gene set, and the functional annotation of the coding genes, are missing in the manuscript.

Are sufficient details of methods and materials provided to allow replication by others?

Partly

Is the rationale for creating the dataset(s) clearly described?

Yes

Are the datasets clearly presented in a useable and accessible format?

Yes

Are the protocols appropriate and is the work technically sound?

Yes

Reviewer Expertise:

Genome assembly, Evolutionary Genomics

I confirm that I have read this submission and believe that I have an appropriate level of expertise to confirm that it is of an acceptable scientific standard, however I have significant reservations, as outlined above.

# Reviewer report: Reviewer response for version 1

**DOI:** 10.21956/wellcomeopenres.25497.r126795

**Published:** 2025-11-03

**Authors:** Nunzio D’Agostino

**Affiliations:** 1 University of Naples Federico II, Piazza Carlo di Borbone, Portici, Italy

Christenhusz et al. present the first genome assembly of Parietaria judaica (pellitory-of-the-wall), a perennial herb widespread in Europe and surrounding regions. The genome is assembled into 13 chromosomal pseudomolecules providing a valuable resource for allergen-related immunological research. The manuscript is well written, with clear objectives, thoroughly documented methods and materials, and a strong experimental design.

 I have only a few minor suggestions to further improve the manuscript: Overall, these minor revisions will strengthen the manuscript and its impact.

 Referring to 'three mitochondrial genomes' is not accurate, since an organism typically has a single mitochondrial genome. It's better to say 'a mitochondrial genome divided into three molecules' or 'a multipartite mitochondrial genome consisting of three distinct DNA molecules.

 "We present the first complete genome of..." Given that the sequencing is not T2T, I think it is more accurate to avoid calling it a 'complete genome’. Indeed ypur assembly covers approximately 72% of the expected genome size (750 Mb).

 Figure 2. There appears to be an issue with the BUSCO ring chart, as the percentages add up to more than 100%, which suggests a potential error. Could you please verify the values? The chart should display the following values: single-copy = 12.3%, duplicated = 83.4%.

 I strongly recommend depositing the cytoplasmic genomes, along with their corresponding annotations, to the NCBI Organelle Genome Resources database

 Please include a dedicated section that clearly outlines the potential for reuse of the dataset presented.

Are sufficient details of methods and materials provided to allow replication by others?

Yes

Is the rationale for creating the dataset(s) clearly described?

Yes

Are the datasets clearly presented in a useable and accessible format?

Partly

Are the protocols appropriate and is the work technically sound?

Yes

Reviewer Expertise:

Genomics and Bioinformatics

I confirm that I have read this submission and believe that I have an appropriate level of expertise to confirm that it is of an acceptable scientific standard, however I have significant reservations, as outlined above.

# Reviewer report: Reviewer response for version 1

**DOI:** 10.21956/wellcomeopenres.25497.r133803

**Published:** 2025-10-30

**Authors:** Oliver Rupp

**Affiliations:** 1 Justus-Liebig-University, Giessengiessen, Germany

This article presents the sequencing, assembly and annotation of the *Parietaria judaica* genome.

 The described data creation and analyses methods are state-of-the art for plant genome assemblies, including PacBio Hifi, Hi-C sequencing for the assembly and RNA-seq to support the gene prediction. The assembly was carried out with hifiasm followed by automatic and manual assembly curation. The assembly quality was assessed by k-mer and orthology based methods like Merqury.FK or BUSCO. 

 The assembly quality metrics are computed on the haploid assembly consisting of 13 chromosomes. The BUSCO metrics identify a high number of duplicated genes (> 80%) and the Hi-C contact map (Figure 5) shows a synteny between chromosomes (e.g. between chromosomes 1 and 3). This should be discussed in the genome report paragraph. This could be an indication of a tetraploid genome. The assembly metrics of the hap2 assembly could help to confirm this and should be included in the report.

Are sufficient details of methods and materials provided to allow replication by others?

Yes

Is the rationale for creating the dataset(s) clearly described?

Yes

Are the datasets clearly presented in a useable and accessible format?

Yes

Are the protocols appropriate and is the work technically sound?

Yes

Reviewer Expertise:

genome assembly and annotation

I confirm that I have read this submission and believe that I have an appropriate level of expertise to confirm that it is of an acceptable scientific standard, however I have significant reservations, as outlined above.

# Reviewer report: Reviewer response for version 1

**DOI:** 10.21956/wellcomeopenres.25497.r133802

**Published:** 2025-10-28

**Authors:** Michael Hiller

**Affiliations:** 1 Senckenberg Research Institute, Frankfurt, Germany

The data note provides a high-quality assembly of *Parietaria judaica.*

 I have 3 comments / questions: 

 I am confused by "While not fully phased, the assembly deposited is of one haplotype." as I understood that a primary assembly that contains a mix of both haplotypes was constructed (the HiC data was not used in HiFiasm to generate a haplotype-resolved assembly according to the methods). 

 While the QV value of mammalian HiFi assemblies is often >60, the Parietaria QV is 55.2. 

 If a QV of ~55 is normal for plants, it would be great to mention this. Otherwise, I wonder if the assembly team knows the reason for the lower QV?

 I also wonder if there is an explanation for the off-diagonal signal in the HiC plot, e.g. between chrom 1 and 3, or 5 and 6? Not being a plant genome expert, I haven't seen such patterns.

Are sufficient details of methods and materials provided to allow replication by others?

Yes

Is the rationale for creating the dataset(s) clearly described?

Yes

Are the datasets clearly presented in a useable and accessible format?

Yes

Are the protocols appropriate and is the work technically sound?

Yes

Reviewer Expertise:

comparative genomics, genome assembly

I confirm that I have read this submission and believe that I have an appropriate level of expertise to confirm that it is of an acceptable scientific standard.

# Reviewer report: Reviewer response for version 1

**DOI:** 10.21956/wellcomeopenres.25497.r133809

**Published:** 2025-10-10

**Authors:** Peng-Fe Ma

**Affiliations:** 1 Chinese Academy of Sciences, Kunming, China

In this study, the genome sequences of a weed, pellitory-of-the-wall, were sequenced and assembled. This plant is also an important host for several butterfly species. The assembled genome is of high quality at the chromosome-level with the assembly statistics above the Benchmark criteria. I noticed that the proportion of duplicated genes in BUSCO is very high (83.4%), however, this plant might be a diploid based on the chromosome number (2n=26). It is better to give more information about the ploidy level of this species.

Are sufficient details of methods and materials provided to allow replication by others?

Yes

Is the rationale for creating the dataset(s) clearly described?

Yes

Are the datasets clearly presented in a useable and accessible format?

Yes

Are the protocols appropriate and is the work technically sound?

Yes

Reviewer Expertise:

Plant genomics and evolutionary biology.

I confirm that I have read this submission and believe that I have an appropriate level of expertise to confirm that it is of an acceptable scientific standard.

# Reviewer report: Reviewer response for version 1

**DOI:** 10.21956/wellcomeopenres.25497.r133807

**Published:** 2025-10-09

**Authors:** Yedomon Ange Bovys Zoclanclounon

**Affiliations:** 1 Rothamsted Research, Harpenden, England, UK

The study presents the first complete genome assembly of *Parietaria judaica*, offering a valuable resource for future research on plant allergenicity, evolutionary biology, and comparative genomics within the Urticaceae family. The genome is of exceptionally high quality, achieved through PacBio HiFi and Hi-C sequencing, resulting in a chromosome-scale assembly with excellent completeness and accuracy metrics (BUSCO 95.7%; k-mer completeness 99.99%; QV 55.2). The manuscript is well-structured, providing clear and comprehensive summaries of assembly statistics, gene annotation, and sequencing metadata, supported by informative visualizations such as snail plots and Hi-C contact maps. Furthermore, the inclusion of open-access datasets, genome browsers, and detailed metadata ensures reproducibility and transparency. The background section effectively situates the study within the broader biological and biomedical context, emphasizing the ecological importance and allergenic relevance of *P. judaica*.

 To enhance the clarity and overall quality of the manuscript, one critical point needs to be addressed. The BUSCO results (C:95.7% [S:12.3%, D:83.4%], F:0.7%, M:3.6%, n:2,326) indicate a remarkably high proportion of duplicated genes, despite the authors’ initial efforts to remove redundancy during assembly. An 83% duplication rate among single-copy orthologs suggests substantial overrepresentation of genomic content. This could result from factors such as heterozygosity and unmerged haplotypes, polyploidy, assembly artifacts or redundancy, contamination, or a recent whole-genome duplication event.

 It is strongly recommended that the authors clarify whether the high number of duplicated BUSCOs is primarily due to heterozygosity, assembly redundancy, or a polyploidy event, given that contamination and redundancy have been technically ruled out through the “purge_dups” and “blobtoolkit” analyses.

Are sufficient details of methods and materials provided to allow replication by others?

Yes

Is the rationale for creating the dataset(s) clearly described?

Yes

Are the datasets clearly presented in a useable and accessible format?

Yes

Are the protocols appropriate and is the work technically sound?

Yes

Reviewer Expertise:

genome assembly, genome annotation, genome evolution, comparative genomics, multi-omics integration

I confirm that I have read this submission and believe that I have an appropriate level of expertise to confirm that it is of an acceptable scientific standard, however I have significant reservations, as outlined above.

# Reviewer report: Reviewer response for version 1

**DOI:** 10.21956/wellcomeopenres.25497.r107490

**Published:** 2024-11-11

**Authors:** Helena Štorchová

**Affiliations:** 1 Institute of Experimental Botany of the Czech Academy of Sciences, Prague, Czech Republic

The manuscript entitled “The genome sequence of pellitory-of-the-wall, *Parietaria judaica* L. (Urticaceae)” presents the assembly of nuclear genome of *P.judaica *as 13  chromosome-level scaffolds and additional 76 scaffolds representing 1.33%. The BUSCO completeness achieves 95.7%.  All the parameters suggest that the nuclear assembly has a high quality.

 I have the following questions:

1. The genome size was estimated as 750 Mb by flow cytometry, but the total length of assembly is only 583.70 Mb. What can be a reason for this discrepancy  ?
2. The authors assembled mitochondrial genome, split into three chromosomes, by MBG software primarily designed for the assembly of large nuclear genomes from long reads. Plant mitochondrial genomes exhibit frequent intramolecular recombination through large repeats and manual curation of the mitogenome assembly is needed. Can be the three mitochondrial chromosomes combined into a single molecule ? Can authors provide more information about the mitogenome assembly ?

Are sufficient details of methods and materials provided to allow replication by others?

Partly

Is the rationale for creating the dataset(s) clearly described?

Yes

Are the datasets clearly presented in a useable and accessible format?

Yes

Are the protocols appropriate and is the work technically sound?

Yes

Reviewer Expertise:

plant genetic and genomics, plant mitochondria

I confirm that I have read this submission and believe that I have an appropriate level of expertise to confirm that it is of an acceptable scientific standard, however I have significant reservations, as outlined above.
